# Supplementary material for: SEIQRS model analysis and optimal control with two delays
Source: PLoS One. 2025 Jun 4;20(6):e0319417. doi: 10.1371/journal.pone.0319417 (PMC12136457; doi:10.1371/journal.pone.0319417)
Supplement: Attachment — Submitted filename: Response to Reviewers.doc [file pone.0319417.s002.doc]

Dear Reviewers,

Thank you for your valuable feedback on our manuscript regarding SEIQRS Model analysis and optimal control with two delays. We appreciate your positive comments on the theoretical aspects of the article. We have carefully considered the concerns you raised and would like to address them as follows:

Response to Reviewer #1:

Thank you for reviewing the paper. We appreciate your feedback. However, we would like to clarify that the introduction of the first delay in the SEIQRS computer virus propagation model is indeed correct. Similar to biological viruses, contact with an infected person does not necessarily lead to immediate infection. Contact with an infected individual takes some time to enter the incubation period. Therefore, there is a lag effect from the susceptible state to the latent state, which needs to be fully considered.

Regarding the mathematical analysis, we understand that it follows classical principles and techniques. However, we believe that applying these principles to the specific context of computer virus transmission models with delays can provide valuable insights into the dynamics of virus spread. We agree that the analysis may not introduce entirely novel mathematical methods, but it contributes to the understanding and control of computer virus propagation. We appreciate your reconsideration of the proposal and we hope that you will find the revised version of the paper to be more satisfactory. We will address your concerns and incorporate any necessary improvements in the revised manuscript. Thank you for your feedback and valuable input.

Response to Reviewer #2:

We appreciate your review and the points you have raised. We will address each of your concerns in the revised version of the paper:

1、The consideration of nonlinear double-delay SEIQRS computer virus models is motivated by the need to capture the complexities of real-world virus propagation dynamics. Delays play a crucial role in modeling the latent period and the recovery time of infected nodes, which are significant factors in understanding and controlling virus spread.

2、The inclusion of two delays in the model corresponds to the two distinct time intervals: the contact delay from susceptible to exposed state and the time required for an infected node to recover and become susceptible again. We will provide a more detailed explanation in the revised manuscript.

3、The purpose of investigating optimal control of the SEIQRS model is to explore strategies for effectively mitigating virus propagation. Optimal control allows us to determine control measures that minimize the number of infected computers while considering the associated costs. It provides insights into the most efficient allocation of resources to combat computer virus outbreaks.

4、We acknowledge the importance of considering potential real-time applications for computer virus propagation models. In the revised manuscript, we will incorporate numerical simulations based on available real-time applications/data to demonstrate the practical implications of the proposed model.

5、We understand the importance of statistical analysis for the proposed model. In the revised version, we will explore suitable real-time applications and provide relevant statistical analyses to support the validity and applicability of the model.

6、In Section 5, we will provide a detailed justification of how the optimum control parameters contribute to mitigating the virus propagation and include the results of the numerical simulations with the control measures applied.

Response to Reviewer #3:

Thank you for your valuable feedback. We appreciate your suggestions for improving the paper. We will address the following modifications in the revised version:

1、The state transition diagram of the computer virus spreading in the network will be included in the revised version of the paper. This diagram will provide a visual representation of the dynamics of the virus transmission.

2、 The rules of the SEIQRS model will be clearly explained in the revised version. This will ensure that readers have a thorough understanding of the model and its components.

3、The change of each node with respect to time will be studied and discussed in the revised paper. This analysis will provide insights into the temporal behavior of the model and its implications for virus spreading.

4、The phase diagram can provide insights into the dynamics of virus spread, such as the existence of stable equilibrium points, limit cycles, or other patterns of behavior. It allows for visualizing the transitions between different compartments and understanding the long-term behavior of the system. The phase diagram takes different state variables as axes and shows how these variables change over time. The SQR phase diagram will be depicted in the revised version. This diagram will visually illustrate the different phases and states of the system, enhancing the understanding of the model dynamics.

5、The references you provided (https://doi.org/10.1002/mma.5530), (https://doi.org/10.1142/S0129183123500523), (https://doi.org/10.1007/s00521-017-2845-7), and (https://doi.org/10.1016/j.rinp.2022.105891) will be cited in the revised paper. These references will support and complement the existing literature review.

6、 All results, including formulas, tables, figures, and analyses, will be carefully revised to ensure accuracy and correctness. We will thoroughly review the content to eliminate any errors or inconsistencies.

7、The English language will be further polished in the revised version. We will address any typos, punctuation errors, and improve the overall clarity of the paper.

8、Clear definitions of all symbols used will be provided, and equations appearing in the paper will be numerated for ease of reference. The keywords and conclusion will be reformulated to accurately reflect the contents of the paper.

9、In the proposed model, all used parameters will be described explicitly. This will ensure transparency and facilitate understanding for readers.

10、The stability of the positive equilibrium point will be utilized and discussed in the revised paper. This analysis will provide insights into the long-term behavior of the system and its stability properties.

11、The value of the objective function J for different models under different control variables will be tabulated in the revised version. This will allow for a comprehensive comparison and analysis of the model's performance under different scenarios.

| Variable of control $\mu(t)$ | 0.00 | 0.20 | 0.40 | 0.60 | 0.80 | opt |
| --- | --- | --- | --- | --- | --- | --- |
| SIRS^[28]^$J$ | 467.50097 | 299.45854 | 212.89711 | 168.93011 | 148.98951 | 127.44763 |
| SIQRS^[29]^$J$ | 31.15207 | 21.83035 | 21.37881 | 27.28561 | 38.347903 | 18.539095 |
| SEIQRS $J$ | 165.18501 | 77.21131 | 51.45359 | 47.07925 | 53.011392 | 35.062202 |

Finally, thank you for your thorough review and insightful suggestions. We sincerely appreciate your feedback, as it will significantly contribute to enhancing the quality and applicability of our manuscript. We genuinely hope that the reviewers will recognize the efforts made to address their concerns and re-evaluate our article accordingly.

Sincerely,

Lei Zhong

E-mail: [6120210176@mail.jxust.edu.cn](mailto:6120210176@mail.jxust.edu.cn)
