# Supplementary material for: SEIQRS model analysis and optimal control with two delays
Source: PLoS One. 2025 Jun 4;20(6):e0319417. doi: 10.1371/journal.pone.0319417 (PMC12136457; doi:10.1371/journal.pone.0319417)
Supplement: Attachment — Submitted filename: Response to reviewers (2).doc [file pone.0319417.s004.doc]

Dear Dr. Hanna Landenmark,

Thank you very much for your review and suggestions on my paper. I have carefully considered the opinions you have raised and have made corresponding revisions and improvements to the paper.

Regarding your specific requirements, I have done the following:

1. I have carefully proofread the paper to ensure smooth language and correction of errors. At the same time, I have also updated the title and abstract submitted online to ensure they are written entirely in English.

2. I have carefully reviewed and evaluated the references recommended by the previous reviewers. I believe these articles are indeed helpful for my literature, as these few articles have sufficient significance for the research on transmission dynamics. After careful consideration, I have decided to cite these references. I have made corresponding adjustments in the paper.

3. I have uploaded the final version of the paper to the editorial system, including the manuscript without revision marks and the response to the review comments.

I hope that the revised version submitted this time can meet the publication requirements of PLOS ONE. If there are any areas that need further improvement, please let me know. I will make the revisions and resubmit as soon as possible.

Thank you again for your valuable opinions and guidance. I look forward to working with you to finally publish this research in your journal.

Sincerely,

Lei Zhong

E-mail: [6120210176@mail.jxust.edu.cn](mailto:6120210176@mail.jxust.edu.cn)
